# Glutamine Modulates Macrophage Lipotoxicity

**DOI:** 10.3390/nu8040215

**Published:** 2016-04-12

**Authors:** Li He, Kassandra J. Weber, Joel D. Schilling

**Affiliations:** 1 Diabetic Cardiovascular Disease Center, Washington University School of Medicine, St. Louis, MO 63110, USA; Lhe@dom.wustl.edu (L.H.); kweber@wayne.med.edu (K.J.W.); 2 Department of Medicine, Washington University School of Medicine, St. Louis, MO 63110, USA; 3 Department of Pathology and Immunology, Washington University School of Medicine, St. Louis, MO 63110, USA

**Keywords:** lysosome, cell death, metabolism, inflammasome

## Abstract

Obesity and diabetes are associated with excessive inflammation and impaired wound healing. Increasing evidence suggests that macrophage dysfunction is responsible for these inflammatory defects. In the setting of excess nutrients, particularly dietary saturated fatty acids (SFAs), activated macrophages develop lysosome dysfunction, which triggers activation of the NLRP3 inflammasome and cell death. The molecular pathways that connect lipid stress to lysosome pathology are not well understood, but may represent a viable target for therapy. Glutamine uptake is increased in activated macrophages leading us to hypothesize that in the context of excess lipids glutamine metabolism could overwhelm the mitochondria and promote the accumulation of toxic metabolites. To investigate this question we assessed macrophage lipotoxicity in the absence of glutamine using LPS-activated peritoneal macrophages exposed to the SFA palmitate. We found that glutamine deficiency reduced lipid induced lysosome dysfunction, inflammasome activation, and cell death. Under glutamine deficient conditions mTOR activation was decreased and autophagy was enhanced; however, autophagy was dispensable for the rescue phenotype. Rather, glutamine deficiency prevented the suppressive effect of the SFA palmitate on mitochondrial respiration and this phenotype was associated with protection from macrophage cell death. Together, these findings reveal that crosstalk between activation-induced metabolic reprogramming and the nutrient microenvironment can dramatically alter macrophage responses to inflammatory stimuli.

## 1. Introduction

Diabetes and obesity are common metabolic disorders that are associated with macrophage dysfunction. Moreover, pathologic inflammation driven by macrophages is recognized as a contributing factor to the impaired tissue repair responses observed in obese and diabetic patients [1,2,3,4]. Therefore, understanding the mechanisms by which the nutrient microenvironment alters macrophage inflammatory responses is highly relevant to human disease. Individuals with obesity and diabetes have excess circulating triglycerides and free fatty acids (FFAs) leading to ectopic lipid deposition in non-adipose tissues including macrophages [5,6]. Toll-like receptor 4 (TLR4) is an inflammatory receptor expressed at high levels on macrophages, which is activated in response to bacterial infection and/or sterile tissue damage [7,8]. Recently, we demonstrated that activation of macrophage TLR4 in a lipid rich environment triggers lysosome damage, which contributes to NLRP3 inflammasome activation and macrophage cell death [9,10,11]. However, the molecular pathways that precede lysosome pathology are not well understood.

Macrophage activation leads to dramatic reprogramming of cellular metabolism to facilitate ATP generation and the production of macromolecules such as proteins, nucleotides, and lipids [12]. Activation of TLR4 on macrophages triggers an increase in glycolysis with variable effects on fatty acid oxidation (FAO) [13,14]. Glutamine uptake and metabolism is also enhanced in response to TLR4 activation [15]. Although metabolic reprogramming is important for macrophage activation, it remains unclear how changes in the nutrient microenvironment interface with these changes in cellular metabolism to influence cell phenotype. This is particularly relevant in situations of nutrient excess where mitochondria can be presented with energetic substrates in excess of what is needed for ATP generation. In this context, oversupply of substrates and reducing equivalents to the mitochondria has the potential to lead to accumulation of metabolites and reactive oxygen species [16].

Glutamine is a non-essential amino acid that is consumed by growing or activated cells. Following uptake, glutamine can be broken down to produce glutamate and α-ketoglutarate (α-KG), the latter of which enters into the TCA cycle. In addition to feeding energetic pathways glutamine also facilitates the uptake of other amino acids, such as leucine via SLC7A5, and promotes activation of growth kinases such as mTOR [17,18]. In line with these observations, when glutamine concentrations are low, mTOR activation is suppressed, and catabolic processes such as autophagy are enhanced [19]. Therefore, glutamine and its metabolites significantly alter cell stress responses, making this a relevant nutrient to consider in the context of lipid-induced macrophage dysfunction. To date, the role of glutamine in macrophage responses to excess FAs has not been explored.

In light of previous data that TLR4 activation in primary macrophages triggers enhanced glucose and glutamine metabolism in macrophages we hypothesized that excess SFA could lead to metabolic gridlock from nutrient overload. This scenario would be expected to result in the accumulation of toxic metabolites that could mediate lysosome damage. Based on prior evidence that lipid-induced cell death is independent of glucose concentration we focused our analysis on how glutamine influences macrophage lipotoxicity [9]. In the absence of glutamine, macrophage cell death and inflammasome activation in response to excess dietary SFAs was attenuated. We provide evidence that glutamine deficiency attenuates the toxic effects of lipid overload likely through its impact on mitochondrial metabolism.

## 2. Experimental Section

### 2.1. Reagents

Bafilomycin A was from Enzo life sciences (Farmingdale, NY, USA). The α-tubulin antibody, α-actin antibody, α-ketoglutarate, and BPTES were from Sigma Chemical (St. Louis, MO, USA). C968 was from Millipore-EMD-Calbiochem (Bellerica, MA, USA). Phospho- and total S6Kinase and AKT S473 antibodies were from Cell Signaling (Danvers, MA, USA). L-glutamine was from Corning (Manassas, VA, USA). The α-LC3 antibody was from Novus Biologicals (NB100-2220; Littleton, CO, USA). The α-p62 antibody was from Abcam (ab56416; Cambridge, MA USA). Lysotracker red was from Life Technologies (Carlsbad, CA, USA). Ultrapure *E. coli* LPS was from Invivogen (San Diego, CA, USA). Thioglycollate was from Difco-BD (Franklin Lakes, NJ, USA). Fatty acids were from Nu-Chek Prep (Waterville, MN, USA). Ultrapure-bovine serum albumin (BSA) was from Lampire (Ottsville, PA, USA) and was tested for TLR ligand contamination prior to use by treating primary macrophages and assaying for TNFα release.

### 2.2. Cell Culture

Peritoneal macrophages were isolated from C57BL/6, or the indicated knockout mice 4 days after intraperitoneal injection of 1 mL, 3.85% thioglycollate and plated at a density of 1 × 10^6^ cells/mL in DMEM containing 10% inactivated fetal serum (IFS), 50 U/mL penicillin G sodium, and 50U/mL streptomycin sulfate (pen-strep). For glutamine free experiments media was prepared with dialyzed serum (Gibco-ThermoFisher, Waltham, MA, USA) to remove all sources of glutamine. Stimulations were performed on the day after harvest. For flow cytometry experiments, peritoneal cells were cultured on low adherence plates (Greiner Bio-One) to facilitate cell harvest. Cells were removed from low adherence plates by washing with PBS followed by 10 min with Cell Stripper (Gibco) and then 10 min with EDTA/trypsin (Sigma). Growth medium was supplemented with palmitate or stearate complexed to BSA at a 2:1 molar ratio, as described previously [9], and BSA-supplemented media was used as control. For cell stimulations, PBS or LPS (100 ng/mL) were added to BSA or free fatty acid containing media.

### 2.3. Mice

Wild type (WT) C57BL/6 mice were bred in our mouse facility; ATG5flox X LysM-Cre, were from Skip Virgin (Washington University). All lines were in the C57BL/6 background. Mice were maintained in a pathogen free facility on a standard chow diet ad libitum (6% fat). All animal experiments were conducted in strict accordance with NIH guidelines for humane treatment of animals and were reviewed by the Animal Studies Committee of Washington University School of Medicine.

### 2.4. RNA Isolation and Quantitative RT-PCR

Total cellular RNA was isolated using Qiagen RNeasy columns and reverse transcribed using a high capacity cDNA reverse transcription kit (Applied Biosystems, Thermo-Fisher Scientific, Waltham, MA, USA). Real-time qRT-PCR was performed using SYBR green reagent (Applied Biosystems) on an ABI 7500 fast thermocycler. Relative gene expression was determined using the delta-delta CT method normalized to 36B4 expression. Mouse primers sequences were as follows (all 5′-3′): *36B4* (forward- ATC CCT GAC GCA CCG CCG TGA, reverse-TGC ATC TGC TTG GAG CCC ACG TT); *LC3* (forward-CGT CCT GGA CAA GAC CAA GT, reverse-ATT GCT GTC CCG AAT GTC TC); *p62* (forward-GCT GCC CTA TAC CCA CAT CT, reverse-CGC CTT CAT CCG AGA AAC).

### 2.5. Western Blotting

Total cellular protein was isolated by lysing cells in 150 mM NaCl, 10 mM Tris (pH 8), triton X-100 1% and 1X Protease Complete and phosphatase inhibitors (Thermo-Fisher Scientific). Subsequently, 25 μg of protein from each sample was separated on a TGX gradient gel (4%–20%; Biorad) and transferred to a nitrocellulose membrane. For blots of Phospho-S6, S6K, and AKT transfer was for 1 h on ice. For LC3 blots, proteins were transferred overnight in the cold room at 140 mAmp constant current.

### 2.6. Lysosome Imaging

After the indicated stimulations, cells were stained with 500 nM lysotracker red in tissue culture media for 15 min at 37 °C. After staining, cells were washed three times with PBS, harvested as described above, and analyzed by flow cytometry.

### 2.7. Metabolism Assays

Cells were plated into 96 well Seahorse plates at density of 75,000 cells/well and stimulated as indicated in the text. After stimulation the cells were washed and placed in XF media (non-buffered RPMI 1640 containing, 25 mM glucose, 2 mM L-glutamine and 1 mM sodium pyruvate) with 10% FCS. Oxygen consumption rates (OCR) and extracellular acidification rates (ECAR) were measured under basal conditions and following the addition the following drugs: 1.5 μM flurorcarbonyl cynade phenylhydrazon (FCCP), and 100 nM rotenone + 1 μM antimycin A (all Sigma). Measurements were taken using a 96 well Extracellular Flux Analyzer (Seahorse Bioscience; North Bellerica, MA, USA).

### 2.8. Ammonia Quantification

After stimulation, supernatants were collected from 0.5 × 10^6^ pMACs macrophages grown in a 24 well plate. The ammonia concentration was determined using an ammonia assay kit (BioVision, Milpitas, CA, USA) as per the manufacturer’s instructions.

### 2.9. Intracellular Glutamine Quantification

Two million pMACs were grown in 6 well plates. After stimulation the cells were washed 3 times with PBS and then were snap frozen and scraped in liquid nitrogen. Intracellular metabolites were quantified by LC-MS/MS at Sanford Burnham Metabolomics Core, Medical Discovery Institute (Lake Nona, FL, USA).

## 3. Results

### 3.1. Glutamine Deficiency Attenuates Macrophage Lipotoxic Responses

**Figure 1:**
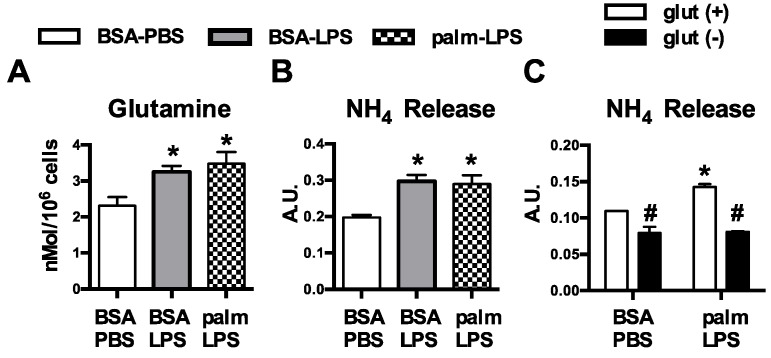
Glutamine metabolism is increased in activated macrophages. (**A**) Peritoneal macrophages (pMACs) were treated with control (BSA-PBS), BSA-LPS (100 ng), or palm (250 µM)-LPS (100 ng) for 16 h; and intracellular glutamine levels were quantified by mass spectroscopy; (**B**) After the indicated stimulations for 16 h, NH_4_ release was quantified in the supernatant; (**C**) pMACs were stimulated in glutamine sufficient (open bars) or glutamine deficient (filled bars) media and NH_4_ release into the media was quantified at 16 h. Bar graphs report the mean ± standard error (SE) for a minimum of 3 experiments, each performed in triplicate. *, *p* < 0.05 for PBS *vs*. LPS; #, *p* < 0.05 glutamine *vs*. no glutamine.

We have previously shown that resting macrophages are largely resistant to the effects of SFA excess; however, upon activation exposure to SFAs leads to lysosome damage, cell death, and inflammasome activation [9,11]. An important consequence of macrophage activation is dramatic reprogramming of cellular metabolism, raising the intriguing possibility that interplay between the internal and external metabolic milieu might be relevant to the toxic effects of lipids. Glutamine metabolism is modulated by TLR4-activation, but the influence of this nutrient pathway on lipid stress responses has not been explored. Consistent with increased uptake of glutamine, we observed that macrophages treated with LPS or LPS with palmitate had increased intracellular glutamine levels (Figure 1A). To further investigate glutamine handling in activated macrophages we quantified ammonia release, a byproduct of glutamine catabolism. Treatment with LPS increased release of ammonia from macrophages under control and lipid stress conditions. In contrast, baseline and LPS-induced ammonia release were significantly decreased when cells were activated in glutamine free conditions (Figure 1B,C).

**Figure 2:**
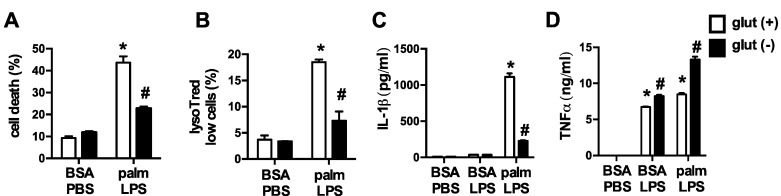
Glutamine deficiency protects against lipotoxicity in macrophages. (**A**,**B**) pMACs were stimulated with BSA-PBS or palm-LPS in glutamine sufficient (open bars) or glutamine deficient (black bars) media and cell death was assessed at 30 h by annexin-PI (**A**), or lysosome damage was determined at 24 h by lysotracker red staining (**B**), both coupled with flow cytometry; (**C**,**D**) After the indicated stimulations IL-1β (**C**) or TNFα (**D**) release was quantified in the supernatant using ELISA. Bar graphs report the mean ± SE for a minimum of 3 experiments, each performed in triplicate. *, *p* < 0.05 for PBS *vs.* LPS; #, *p* < 0.05 glutamine *vs*. no glutamine.

To elucidate the impact of glutamine on lipid-induced macrophage lysosome damage we activated pMACs with palmitate and LPS in control media or media deficient in glutamine. In the absence of glutamine, macrophage cell death and lysosome damage in response to palmitate and LPS were significantly decreased (Figure 2A,B). In addition, release of the inflammasome regulated cytokine IL-1β was also diminished when glutamine was absent. TNFα secretion, which is not regulated by the inflammasome or lysosome damage, was not reduced with glutamine deficiency indicating that macrophage inflammatory function was not globally suppressed (Figure 2C,D). Thus, alterations in TLR4 activation-induced glutamine metabolism are required for FAs in the nutrient microenvironment to produce toxicity in macrophages.

### 3.2. Oxidative Glutamine Metabolism Is Partially Responsible for the Protection from Lipid Toxicity

**Figure 3:**
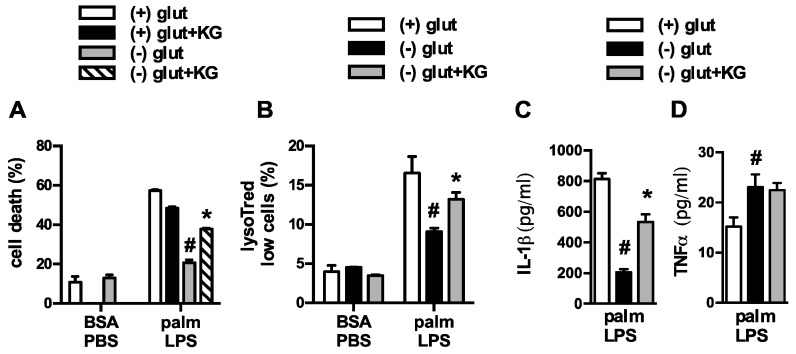
α-ketoglutarate partially restores macrophage lipotoxic phenotypes under glutamine deficient conditions. (**A**) Macrophages were treated as indicated in glutamine sufficient and deficient media ± 440 nM α-ketoglutarate (α-KG) and cell death (**A**) or lysotracker low cells (**B**) were quantified by flow cytometry; (**C**,**D**) pMACs were stimulated with palm-LPS in glutamine sufficient and deficient media ± α-KG and IL-1β (**C**) or TNF α (**D**) release was quantified by ELISA. Bar graphs report the mean ± SE for a minimum of 3 experiments, each performed in triplicate. *, *p* < 0.05 for veh *vs*. α-KG; #, *p* < 0.05 glutamine *vs*. no glutamine.

**Figure 4:**
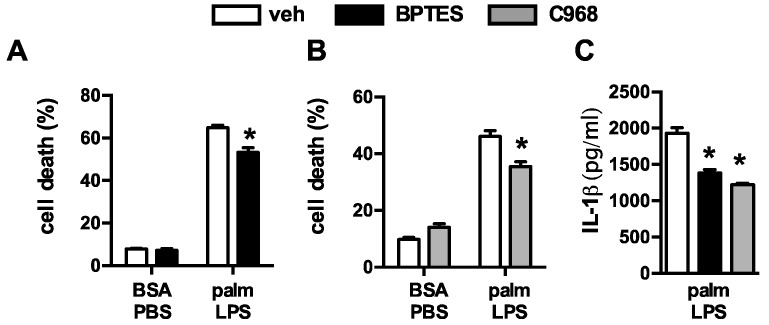
Inhibition of glutaminolysis partially mimics glutamine deficiency. (**A**,**B**) Primary macrophages were stimulated with BSA-PBS in the presece of the glutaminolysis inhibitors BPTES ((**A**) 10 µM) or C698 ((**B**) 10 µM); (**C**) IL-1β release was quantified from pMACs treated with palm-LPS in the presence of BTPES of C968. Bar graphs report the mean ± SE for a minimum of 3 experiments, each performed in triplicate. *, *p* < 0.05 for veh *vs*. inhibitor.

Upon entering the cell, glutamine can be broken down by glutaminase to glutamate, which is then converted to α-ketoglutarate (α-KG) for oxidation in the TCA cycle. To determine whether depletion of α-KG was the cause of reduced macrophage lipotoxicity we added a membrane permeable form of α-KG to glutamine deficient or sufficient media and assessed the lipotoxic phenotypes. Restoration of α-KG significantly increased cell death and lysosome damage in macrophages stimulated in glutamine deficient media (Figure 3A,B). In contrast, the addition of α-KG to macrophages cultured in glutamine sufficient media did not increase macrophage cell death. Similar findings were observed for IL-1β release (Figure 3C,D). However, the lysosome damage and cell death observed with α-KG supplementation was still less severe compared to glutamine sufficient media, arguing that some of the effects of glutamine are independent of glutamine breakdown. To further address this issue we used two chemically distinct inhibitors of glutaminase, Bis-2-(5-phenylacetamido-1,3,4-thiadiazol-2-yl)ethyl sulfide (BPTES) or compound 968. With both agents cell death and IL-1β release were attenuated, although the compounds were significantly less effective than glutamine deprivation (Figure 4A–C). These findings are consistent with the α-KG add-back experiments, and suggest that glutamine deprivation protects through pathways that are both dependent and independent of its catabolism.

### 3.3. Glutamine Deficiency Is Protective Independent of Leucine

**Figure 5:**
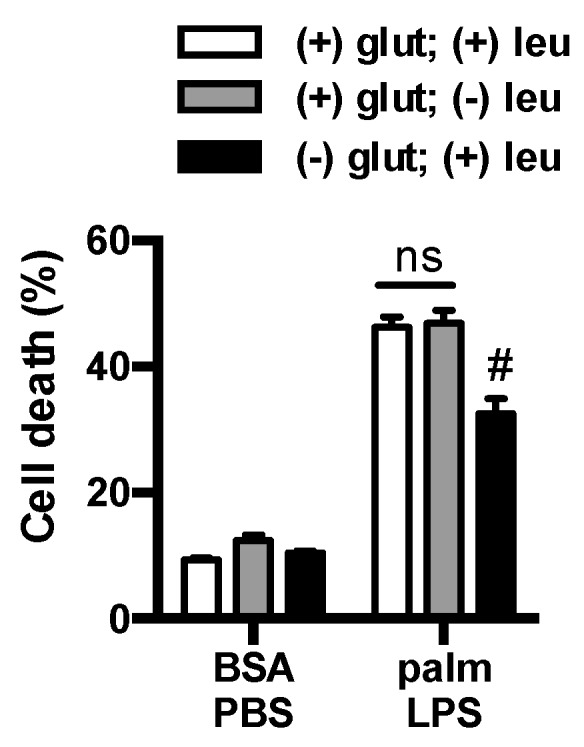
Leucine deficiency does not protect macrophage from lipotoxicity. Macrophages were incubated in complete RPMI media (open bars) or RPMI lacking leucine (gray filled bars) or glutamine (black filled bars), and after the indicated stimulation cell death was determined by annexin-PI flow cytometry. Bar graphs report the mean ± SE for a minimum of 3 experiments, each performed in triplicate. #, *p* < 0.05 glutamine *vs*. no glutamine.

Glutamine is required for the cellular import of branched chain amino acids such as leucine [20]. Thus, a possible catabolism-independent function of glutamine deprivation could be explained by reduced leucine uptake. To investigate whether leucine deprivation could mimic the effects of glutamine removal we used media deficient in glutamine or leucine and analyzed macrophage cell death. Removal of leucine from glutamine containing media did not protect the macrophages from cell death (Figure 5). Thus, the phenotype of glutamine deficiency cannot be explained by reduced uptake and metabolism of leucine.

### 3.4. mTOR Signaling Is Reduced in the Absence of Glutamine

**Figure 6:**
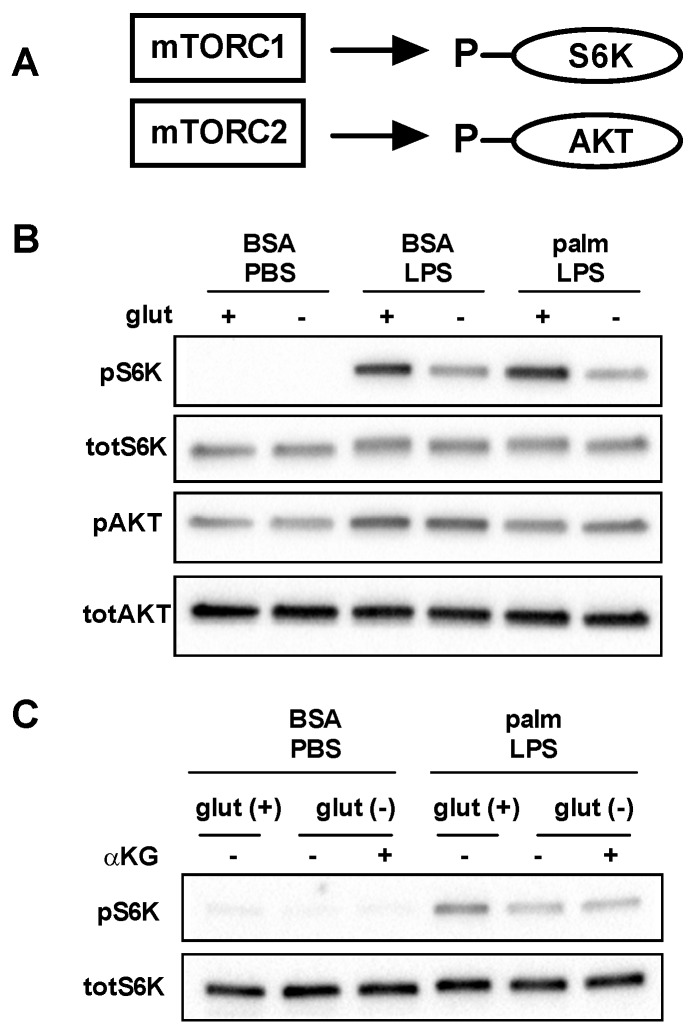
Glutamine deficiency impairs activation of mTORC1. (**A**) mTORC can assemble in two distinct complexes known as mTORC1 and mTORC2 which lead to the phosphorylation of S6K and AKT, respectively; (**B**) pMACs were stimulated with BSA-PBS, BSA-LPS, or palm-LPS for 16 h in the presence or absence of glutamine and phosphorlyation of S6K and AKT were assessed by Western blotting. Total S6K and total AKT are shown as control; (**C**) Macrophages were treated as indicated for 16 h in glutamine sufficient or deficient media ± α-KG (440 nM) and S6K phosphorylation was determined by Western blotting.

mTOR is a nutrient responsive kinase that is activated by growth factors and/or amino acids, including glutamine. mTOR exists as two complexes known as mTORC1 and mTORC2 in which mTOR pairs with the adaptor proteins raptor or rictor, respectively (Figure 6A) [21]. To assess activation of these mTOR complexes in the presence and absence of glutamine we analyzed phosphorylation of the mTORC1 substrate S6kinase (S6K) and the mTORC2 substrate AKT. Consistent with prior reports, LPS led to increased phosphorylation of both S6K and AKT. In the absence of glutamine S6K phosphorylation was impaired, but no effect was observed on AKT phosphorylation. In contrast, the SFA palmitate reduced AKT phosphorylation, but did not significantly affect S6K phosphorylation (Figure 6B). We next determined whether the addition of α-KG could restore activation of S6K. As can be seen in Figure 6C, α-KG add-back did not increase S6K phosphorylation in the absence of glutamine, suggesting this was a direct effect of glutamine on mTORC1 activation and less likely to be involved in the protection from lipotoxicity.

### 3.5. Glutamine Deficiency Modulates Macrophage Autophagy

**Figure 7:**
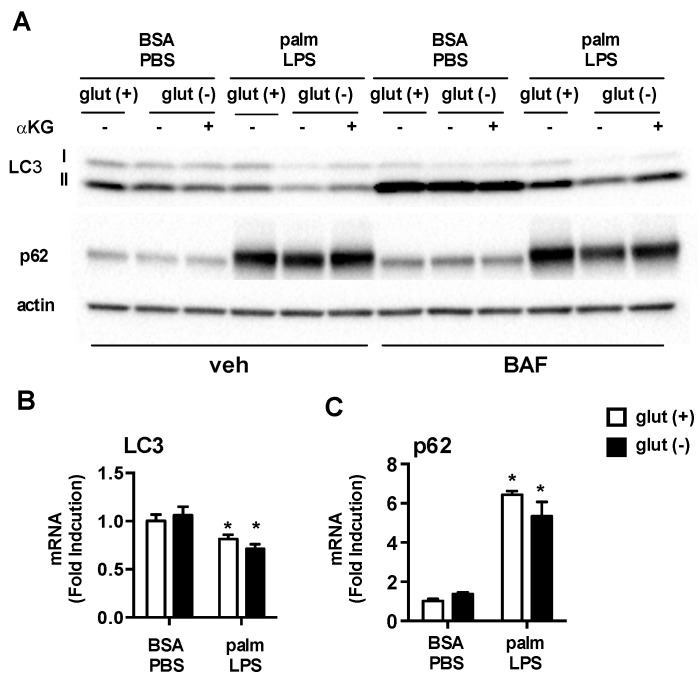
Autophagy is modulated by glutamine deficiency. (**A**) pMACs were treated with BSA-PBS or palm-LPS in glutamine sufficient or glutamine deficient media ± α-KG (440 nM). The cells were incubated for 16 h followed by 2 h of veh or bafilomycin (BAF; 50 nM). Protein lysates were analyzed for expression of LC3 or p62. Actin is shown as a loading control; (**B**) Cells were stimulated as indicated for 16 h in media with or without glutamine followed by qRT-PCR assessment of LC3 and p62 mRNA expression. Bar graphs report the mean ± SE for a minimum of 3 experiments, each performed in triplicate. *, *p* < 0.05 for PBS *vs*. LPS; #, *p* < 0.05 glutamine *vs*. no glutamine.

One important function of mTORC1 is to regulate autophagy, a fundamental process involved the starvation response and in cell quality control. Glutamine availability has also been implicated as a regulator of autophagy. Thus, to monitor autophagic flux in our system we performed western blots of the autophagy proteins LC3 and p62 with or without bafilomycin present for the last 2 h of the stimulation to block lysosomal flux. Under control conditions with BSA-PBS there were no differences in LC3II or p62 protein levels whether or not glutamine was present. Treatment with bafilomycin to block lysosomal degradation of autophagosomes increased both LC3II and p62 consistent with active autophagic flux in these macrophages. Again, glutamine deficiency did not alter autophagic flux under control conditions. In contrast, the absence of glutamine in cell treated with palm-LPS led to decreased protein levels of LC3II and p62 and this response was partially restored by α-KG. In bafilomycin treated samples LC3II levels increased in all of the treatment groups, although the greatest increase was observed for BSA-PBS treated samples (Figure 7A). These findings are consistent with reduced flux through the lysosome under lipid stress conditions. Even in the presence of bafilomycin, LC3II levels were still reduced in palm-LPS samples from glutamine free conditions. As LC3 and p62 are also regulated at the transcriptional level we assessed whether the observed differences between glutamine sufficient or deficient conditions were driven by transcriptional changes using qRT-PCR. Treatment with palm-LPS decreased LC3 and increased p62 mRNA levels compared to control, but glutamine status did not alter this profile (Figure 7B). Together this data reveals that under lipid stress conditions the absence of glutamine results in reduced LC3II protein likely through a combination of increased autophagic degradation together with another post-translational regulatory pathway, perhaps autophagolysosomal exocytosis [22].

**Figure 8:**
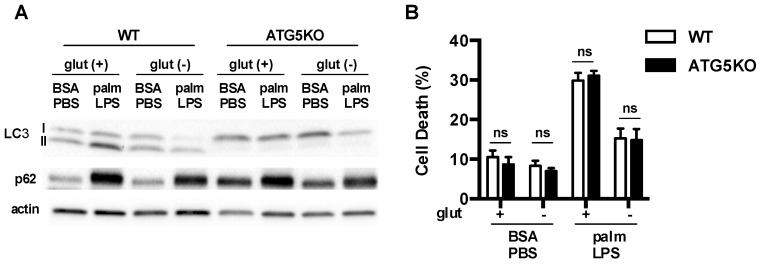
Autophagy is dispensable for protective effects of glutamine deficiency. (**A**) Primary macrophages were isolated from WT (ATG5 flx/flx) or ATG5KO (ATG5 flx/flx-LysM-Cre) followed by stimulation with BSA-PBS or palm-PLS in the presense or absence of glutamine. The levels of LC3 and p62 protein protein expression were assessed by western blotting. Actin is shown as a protein loading control; (**B**) pMACs from WT and ATG5KO mice were stimulated as indicated and cell death was determinded at 30 h using annexin-PI flow cytometry. The bar graph reports the mean ± SE for a minimum of 3 experiments, each performed in triplicate. *, *p* < 0.05 for WT *vs*. ATG5KO.

To determine whether augmentation of macrophage autophagy under glutamine free conditions was required for the protection against lipid stress, we turned to a model of autophagy deficiency. ATG5 is absolutely required for classical autophagy. Therefore, we utilized macrophages in which ATG5 had been knocked out using a myeloid specific Cre driver (lysM-Cre). In ATG5 knockout (KO) macrophages we observed that LC3 was predominantly in the non-lipidated form known as LC3I and p62 levels were increased at baseline (Figure 8A). These observations indicate a block at the initiation step of autophagy. Wild type (WT) and ATG5KO macrophages were then activated in the presence or absence of the FA palmitate ± glutamine. As we had shown previously, palm-LPS induced macrophage cell death was not affected by ATG5 deficiency [9]. Importantly, the protection afforded by glutamine deficiency occurred in both WT and ATG5KO macrophages, demonstrating that this rescue phenomenon is independent of autophagy (Figure 8B).

### 3.6. Glutamine Deficiency Prevents the Suppression of Mitochondrial Function by Palmitate

**Figure 9:**
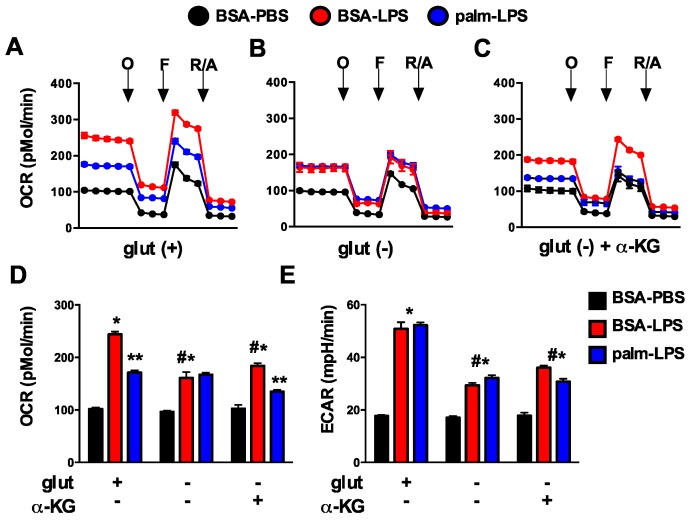
Glutamine deficiency alters mitochondrial metabolism. (**A**–**C**) pMACs were stimulated for 16 h with BSA-PBS (black), BSA-LPS (red), or palm-LPS (blue) in glutamine sufficient media (**A**) or glutamine deficient media ± α-KG (**B**,**C**) and mitochondrial metabolism was assessed using a Seahorse flux analyzer. Mitochondrial oxygen consumption rate (OCR) was assessed at baseline and after the injection of oligomycin (**O**), FCCF (**F**) and rotenone/antamycin (R/A); (**D**,**E**) Baesline OCR (**D**) or extracellular acidification rate (ECAR; (**E**)) are reported from cells stimulated as in (**A**–**C**). Bar graphs report the mean ± SE for a minimum of 3 experiments, each performed in triplicate. *, *p* < 0.05 for BSA-PBS *vs*. BSA-LPS; **, *p* < 0.05 BSA-LPS *vs*. palm-LPS; #, *p* < 0.05 glutamine *vs*. no glutamine.

Glutamine is a critical substrate for mitochondrial metabolism and TCA function and can directly or indirectly impact mitochondrial metabolism. To investigate the affects of glutamine deficiency on cellular metabolism we employed a Seahorse flux analyzer to measure mitochondrial oxygen consumption rate (OCR) and extracellular acidification rate (ECAR), a marker of glycolytic flux. In response to LPS, macrophage OCR at baseline dramatically increased and this response was suppressed by the presence of palmitate. Maximal OCR, assessed after injection of the uncoupling agent FCCP, was also increased with LPS (Figure 9A). In the absence of glutamine, macrophages had similar baseline OCR under control conditions compared to glutamine sufficient conditions; however, upon activation with LPS the increase in mitochondrial OCR was significantly less than that seen in glutamine sufficient conditions. In addition, palmitate no longer suppressed LPS-induced mitochondrial respiration as it did when glutamine was present. The addition of α-KG to glutamine deficient media yielded only a small increase in LPS-induced OCR, but the suppression of mitochondrial respiration in response to palmitate returned (Figure 9A–D). Glycolytic flux as estimated by ECAR was also reduced in the absence of glutamine, but unlike OCR this response was unaffected by the presence of palmitate. The addition of α-KG to glutamine deficient media led to a very small increase in ECAR (Figure 9E). These results indicate that the toxicity of excess SFA appears to track with mitochondrial respiration dynamics as opposed to changes in ECAR/glycolysis.

## 4. Discussion

It is increasingly being recognized that the nutrient composition present in tissues can influence the phenotypes and inflammatory functions of macrophages [23]. The central finding of this study is that the removal of glutamine from the nutrient microenvironment renders activated macrophages resistant to the toxic effects of dietary SFAs. Consistent with a role for glutaminolysis in this process, the sensitivity to lipid stress is partially restored by the addition of α-KG, an important metabolite of glutamine breakdown. Although mTOR activation and autophagic flux are altered by glutamine deficiency, the mechanism of protection appears to be independent of these pathways. Instead alterations in mitochondrial oxidative metabolism that occur in the absence of glutamine are associated with macrophage sensitivity to cell death. These findings add to a growing body of literature arguing that the interplay between lipid and amino acid metabolism in the mitochondria is relevant to cell dysfunction in metabolic disease [24,25].

Glutamine is the most abundant amino acid in the body and its uptake and utilization are significantly increased in macrophages following activation signals, such as LPS [15]. Once inside the cell glutamine has several potential fates; however, the best-described metabolic pathway is its break down to glutamate and α-KG by the enzymes glutaminase and glutamate dehydrogenase, respectively. The activity of both of these enzymes is enhanced in LPS-activated macrophages [26]. The α-KG generated by these reactions is critical for TCA cycle anapleurosis, particularly since oxidation of citrate is reduced by LPS activation [27]. However, the majority of glutamine does not undergo complete oxidation and instead is converted to ammonia, aspartate, or pyruvate/lactate [28]. To determine the importance of glutaminolysis in cell death we used two approaches. First, we added back the glutaminolysis product α-KG to cells lacking glutamine and discovered a partial restoration of lipotoxicity. We also inhibited glutaminolysis using two chemically distinct inhibitors of glutaminase. Both of these approaches suggested that glutamine catabolic pathways were partially responsible for the observed effects on lipotoxicity. We also considered that an important non-catabolic function of glutamine might be related to its role in the import of leucine via SLC7A5 [18]. However, cell death was not affected under leucine free conditions arguing that impaired uptake of this branched chain amino acid did not contribute to the protection afforded by glutamine deficiency.

Glutamine directly and indirectly modulates pathways related to the kinase mTOR and autophagy [17,20]. In this study we showed that removal of glutamine from the nutrient microenvironment during LPS activation modestly decreased the activity of mTORC1 as assessed by the phosphorylation status of S6K. This phenotype was specific to mTORC1 as the mTORC2 substrate AKT was unaffected under these conditions. However, the addition of α-KG to glutamine deficient media did not restore mTOR activation, suggesting that suppression of mTORC1 was not the primary mechanism accounting for reduced lipotoxicity. Autophagy is also regulated by glutamine, where glutamine deficiency increases flux through this pathway [29]. Reduced mTORC1 activity is at part of this response; however, decreased levels of other metabolites, such as cytosolic acetyl-CoA, may also play a role in increasing autophagy [17,30]. In other systems autophagy has been shown to protect against cell death related to accumulation of damaged organelles and proteins [31,32]. We show that in macrophages the absence of glutamine did not change autophagic flux at baseline; however, after LPS activation autophagy rates were increased in glutamine deficient macrophages. However, genetic disruption of autophagy using ATG5KO macrophages did not reverse the protection afforded by lack of glutamine. Thus, although mTORC1 activity is reduced and autophagy is enhanced in a glutamine-free nutrient microenvironment, the protection from lipotoxicity occurs independent of these events.

As discussed above, glutamine is an important contributor to mitochondrial metabolism in part through the replenishment of TCA cycle intermediates. Using functional metabolism studies we discovered an interesting mitochondrial phenotype in LPS activated cells lacking glutamine. As expected, the increase in mitochondrial OCR induced by LPS activation was substantially attenuated in the absence of glutamine. Interestingly, addition of α-KG to glutamine deficient media did not reverse this OCR phenotype indicating that the mitochondrial reprograming is more than just a loss of TCA cycle flux. The most striking difference was that the suppressive effect of palmitate on LPS-induced OCR was reversed in glutamine free media and partially restored with addition of α-KG. Although glycolytic flux, as estimated by ECAR, was also suppressed by ~50% in glutamine deficient media neither palmitate nor α-KG significantly changed this phenotype.

**Figure 10:**
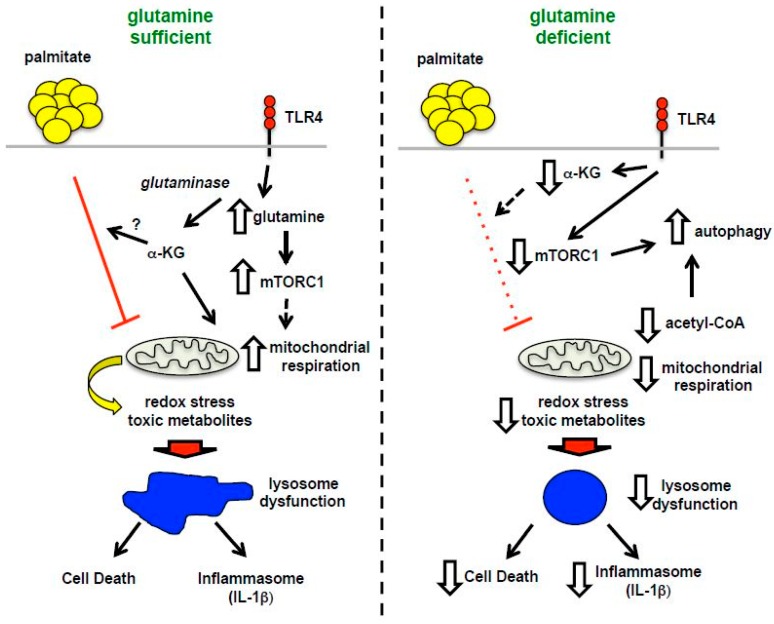
Model of macrophage lipotoxicity under glutamine sufficient and deficient conditions. When glutamine is available, TLR4 activation stimulates glutamine uptake, mTORC1 activation, enhanced formation of TCA intermediates, such as α-KG, and increased mitochondrial respiration. The presence of excess fatty acids like palmitate leads to suppression of mitochondrial respiration, which in the setting of LPS activation likely promotes accumulation of toxic metabolites, lipids and/or redox stress. Lysosome dysfunction ensues leading to macrophage cell death and inflammasome activation. In the absence of glutamine, mTORC1 activation and mitochondrial respiration are diminished leading to enhanced rates of autophagy. In this scenario palmitate no longer suppresses mitochondrial function, which appears to protect against lysosome dysfunction, perhaps by decreasing the formation of damaging metabolites and lipids.

It is now appreciated that obesity and diabetes impose a multifaceted nutrient stress on the mitochondria. Specifically, the excess delivery of an uptake of FAs and amino acid metabolites to the mitochondria can produce metabolic gridlock, especially when energy stores are replete [16]. In this context, a “back pressure” develops on the mitochondrial electron transport chain due to lower activity of ATP synthase. The consequence of this backlog can be the generation of reactive oxygen species and/or accumulation of other metabolites, which have the potential to damage proteins and organelles. Our data suggest that glutamine deficiency reduces the mitochondrial substrate burden, which may be protective against cell damage in the setting of lipid overload. As we have previously shown that scavenging of ROS does not reduce lysosome damage or cell death in our system, we expect other pathways connecting mitochondrial metabolites (acetyl-CoA, acylcarnitines, etc.) with lysosome damage exist [9] (Figure 10). This is an area of active exploration.

Our findings may be relevant to several physiologic situations in obese and diabetic hosts. In the context of acute infection or tissue injury glutamine is released from skeletal muscle and its uptake by macrophages is enhanced [33]. When excess lipids are present, as in diabetes, macrophage inflammasome activation and cell death are anticipated to increase, contributing to the persistent inflammatory response seen in diabetics. The potential relevance of this pathway is supported by recent data revealing that the inflammasome is a major contributor to diabetic inflammation *in vivo*, including in humans [34,35,36]. In addition to acute inflammation, diabetes is associated with a chronic inflammatory response [37]. Macrophages are recruited to adipose tissue during obesity and are thought to contribute to the development of systemic insulin resistance [38,39]. It is well established that macrophages accumulate lipid in obese adipose tissue suggesting that interaction between FAs and glutamine metabolism would be of interest to explore in this situation [40]. Such studies could be performed using macrophage specific knockout of glutamine transporters. Further investigation into the role of glutamine in other inflammatory conditions that often co-exist with diabetes such as vascular and rheumatologic disease could also be of interest.

There are several limitations of the current study that must be acknowledged. In order to perturb the nutrient microenvironment, we used an *in vitro* system with thioglycollate-elicited peritoneal cells as a model of monocyte-derived macrophages. Therefore, *in vivo* approaches will be necessary to determine the relevance of these findings to diabetic inflammation. Moreover, the studies were performed with murine macrophages and therefore additional studies with human monocyte-derived macrophages are warranted. It is also important to acknowledge that glutamine uptake by immune cells is important for full activation and therefore deprivation of this nutrient could lead to increased risk of infection [26]. The relative balance of risk and benefit in diabetic patients will require further investigation.

Macrophage dysfunction is a hallmark of lipid overload disorders such as obesity and diabetes. In response to excess dietary saturated FAs activated macrophages develop lysosome damage, inflammasome activation, and cell death and these events appear to be involved in clinical complications of these metabolic conditions [3,36,41,42,43]. However, the mechanisms that lead to these phenotypes are not well understood. Herein we describe an intriguing example whereby TLR4-induced metabolic reprogramming of glutamine metabolism can be maladaptive in the setting of lipid overload. These findings suggest that nutritional or pharmacologic interventions could potentially be used to modulate macrophage function for therapeutic benefit in metabolic disease.
